# Age at Initiation of Cigarette Use in a Nationally Representative Sample of US Youth, 2013-2017

**DOI:** 10.1001/jamanetworkopen.2021.0218

**Published:** 2021-02-26

**Authors:** Adriana Pérez, Roi San N’hpang, Elizabeth Callahan, Meagan Bluestein, Arnold E. Kuk, Baojiang Chen, Cheryl L. Perry, Melissa B. Harrell

**Affiliations:** 1 Department of Biostatistics and Data Science, The University of Texas Health Science Center at Houston, School of Public Health, Austin; 2 Michael & Susan Dell Center for Healthy Living, The University of Texas Health Science Center at Houston, School of Public Health, Austin; 3 Department of Epidemiology, Human Genetics and Environmental Sciences, The University of Texas Health Science Center at Houston, School of Public Health, Austin; 4 Department of Health Promotion and Behavioral Sciences, The University of Texas Health Science Center at Houston, School of Public Health, Austin

## Abstract

**Importance:**

Cigarettes are still a commonly used tobacco product among youth despite recent declines in cigarette use.

**Objective:**

The aim of this study was to prospectively estimate the age of cigarette use initiation among youth (aged 12-17 years) overall, by sex, and by race/ethnicity.

**Design, Setting, and Participants:**

This cohort study used data from waves 1 through 4 of the nationally representative Population Assessment of Tobacco and Health (PATH) study, conducted from September 12, 2013, to January 3, 2018. Two subpopulations of youth were assessed: (1) those nonsusceptible to cigarette use and (2) never users of cigarettes at their first wave of PATH participation. Weighted interval-censoring survival analyses were used to prospectively estimate the age of initiation of cigarette use outcomes. Weighted interval-censoring Cox proportional hazard models were used to estimate differences in the age of initiation by sex and by race/ethnicity. Statistical analyses were performed from October 7, 2019, to May 1, 2020.

**Exposures:**

Differences in the age of initiation by sex and race/ethnicity.

**Main Outcomes and Measures:**

Age of initiation of susceptibility to cigarette use, ever use, past 30-day use, and fairly regular cigarette use overall, by sex, and by race/ethnicity.

**Results:**

A total of 15 776 youth never users and, among them, 11 022 youth who were nonsusceptible to cigarette use, were included in this study (weighted mean [SE] age, 13.5 [0.01] years; 58.6% [SE, 0.46%] non-Hispanic White; and 51.0% [SE, 0.32%] boys) and 15 776 were never users of cigarettes at their first wave of PATH participation (weighted mean [SE] age, 13.7 [0.01] years; 55.0% [SE, 0.29%] non-Hispanic White; and 51.0% [SE, 0.15%] boys). By age 18 years, among those who were nonsusceptible, 46.2% (95% CI, 44.3%-48.2%) became susceptible to cigarette use. Among never users, 24.4% (95% CI, 22.9%-25.9%) initiated ever cigarette use, 16.4% (95% CI, 15.2%-17.6%) initiated past 30-day cigarette use, and 4.3% (95% CI, 3.9%-4.8%) initiated fairly regular cigarette use. Boys had a higher risk of initiating ever (hazard ratio [HR], 1.21; 95% CI, 1.08-1.36) and past 30-day cigarette use (HR, 1.27; 95% CI, 1.10-1.47) at earlier ages compared with girls. Non-Hispanic White youth had a higher risk of an earlier age of initiation of susceptibility to cigarette use (HR, 0.77; 95% CI, 0.68-0.88), ever use (HR, 0.59; 95% CI, 0.49-0.71), past 30-day use (HR, 0.64; 95% CI, 0.52-0.77), and fairly regular cigarette use (HR, 0.25; 95% CI, 0.14-0.43) compared with non-Hispanic Black youth.

**Conclusions and Relevance:**

The results of this cohort study suggest that, despite current interventions and existing laws, a large number of youth initiated cigarette use before the legal age to purchase tobacco products.

## Key Points

**Question:**

At what age do youth (aged 12-17 years) initiate 4 cigarette use outcomes (susceptibility to use, ever use, past 30-day use, and fairly regular use)?

**Findings:**

This cohort study examined nationally representative data for 15 776 youth never users and, among them, 11 022 youth who were nonsusceptible to cigarette use, and found that among youth who were nonsusceptible to cigarette use, by age 18 years, 46.2% became susceptible to cigarette use. Among never users of cigarettes, 24.4% initiated ever cigarette use, 16.4% initiated past 30-day cigarette use, and 4.3% initiated fairly regular cigarette use.

**Meaning:**

Study results suggest that prevention interventions and educating the public about targeting specific ages, particularly before age 17 to 18 years, may reduce cigarette use initiation.

## Teaser

This cohort study uses data from the Population Assessment of Tobacco and Health (PATH) study waves 1 to 4 to assess at what age young people aged 12 to 17 years initiate cigarette use.

## Introduction

Identifying the age of initiation of tobacco use has been pivotal to achieving reductions in tobacco use nationwide, as tobacco use remains one of the leading causes of preventable diseases and death in the US.^1,2^ According to the 2012 Surgeon General’s report, 88.2% of adult daily smokers aged 30 to 39 years recall initiating cigarette use at or before age 18 years.^3^ Despite laws in place enforcing the minimum age of tobacco sales (18 years until December 2019; 21 years since December 2019), it is evident that youth are able to access tobacco products, including cigarettes.

Susceptibility to cigarette smoking has been identified as a risk factor for smoking initiation before youth start using cigarettes.^4,5^ A previous publication of the Population Assessment of Tobacco and Health (PATH) study, conducted from September 12, 2013, to December 14, 2014, reported the prevalence of susceptibility to cigarette use among youth (aged 12-17 years) overall (28.6%)^6^ and by race/ethnicity (for Hispanic youth, 31.3%; for non-Hispanic Black youth, 30.5%; and for non-Hispanic White youth, 26.9%).^6^ The 2019 National Youth Tobacco Survey (NYTS) reported cigarette susceptibility by sex (boys, 46.4%; girls, 45.5%).^7^ In addition, previous reports of PATH among youth have reported initiation of ever (3.8%)^8^ and past 30-day (1.6%)^8^ cigarette use after 1 or 2 years of follow-up,^9^ as well as differences by sex and race/ethnicity. Middle and high school students from the 2014-2016 NYTS reported their median recalled age of cigarette initiation as 13 years, but this finding is prone to recall bias.^10,11,12^

As the popularity of other tobacco products has been increasing among youth in recent years,^13^ nearly all cigarette users initiate before age 18 years,^3^ and earlier ages of cigarette use are associated with increased nicotine dependence^3^ and higher risk for chronic diseases.^14,15^ Therefore, it is necessary to examine the age of cigarette initiation for tobacco control.

Information that has been missing from the previously mentioned studies is the age of cigarette initiation overall, by sex, and by race/ethnicity, estimated prospectively using survival analyses with 4 years of follow-up. Therefore, we conducted a secondary analysis of PATH^16^ prospectively estimating the age of initiation of cigarettes in youth (aged 12-17 years) for (1) susceptibility to cigarette use, (2) ever use, (3) past 30-day use, and (4) fairly regular use among nonsusceptible (1) and never users of cigarettes at their first wave of PATH participation. Fairly regular use is a subjective measure used to identify consistent, committed cigarette use.^17^

## Methods

### Study Design and Participants

PATH is a nationally representative, longitudinal cohort study of US youth and adults that studies tobacco use behaviors, attitudes and beliefs, and tobacco-related health outcomes.^16^ The target population of PATH consisted of individuals 12 years and older across the US, and 13 651 youth (aged 12-17 years) completed wave 1 (September 12, 2013, to December 14, 2014). Our study includes 2 subpopulations: (1) youth aged 12 to 17 years who were nonsusceptible to cigarette use at their first wave of PATH participation (waves 1-3) and (2) youth aged 12 to 17 years who were never cigarette users at their first wave (waves 1-3) of PATH participation. In addition, youth aged 9 to 11 years at wave 1 were considered “aged-up youth” when they turned 12 years old and were eligible to participate in the study at waves 2 to 3; 2091 and 2045 aged-up youth were included in our study at waves 2 (October 23, 2014, to October 30, 2015) and 3 (October 19, 2015, to October 23, 2016), respectively. Youth who turned 18 years of age were invited to complete the adult measurements; 1915, 1907, and 1900 aged-up adults completed the adult questionnaire from waves 2 to 4 (October 23, 2014, to January 3, 2018), respectively. Data from waves 2 to 4 were used to track outcomes for all participants. Informed oral consent was obtained from the parents of the youth participants, and the youth provided oral assent.^16^ The University of Texas Health Center at Houston granted institutional review board approval (HSC-SPH-17-0368). This report followed the Strengthening the Reporting of Observational Studies in Epidemiology (STROBE) reporting guideline for cohort studies.

### Measures

#### Cigarette Outcomes

The following questions were used to measure susceptibility to cigarette use among nonusers of cigarettes across all 4 waves (2013- 2017): (1) “Have you ever been curious about smoking a cigarette?”^18^ (2) “Do you think that you will try a cigarette soon?”^18^ and (3) “If one of your best friends were to offer you a cigarette, would you smoke it?”^18^ Response options for the first question were “very curious,” “somewhat curious,” “a little curious,” and “not at all curious.”^18^ Response options for the next 2 questions were “definitely yes,” “probably yes,” “probably not,” and “definitely not.”^18^ Respondents who answered “not at all curious” to the first question and “definitely not” to the next 2 questions were considered nonsusceptible to cigarette use, and any other combination of responses was considered susceptible to cigarette use.

The PATH study used a derived variable to represent ever cigarette use (yes or no) at each wave.^18^ Past 30-day cigarette use was measured with the question “In the past 30 days, on how many days did you smoke cigarettes?”^18^ Numeric response options included 0 to 30 days, and participants were considered past 30-day cigarette users if they reported 1 or more days. Fairly regular cigarette use (yes or no) was measured with the question “Have you ever smoked cigarettes fairly regularly?”^18^ For all questions, participants who responded “don’t know” or “refused” were excluded from the analysis.

#### Sex and Race/Ethnicity as Exposures

The PATH study imputed the self-reported participant sex variable by using the household information at wave 1 but not at waves 2 and 3^18^ and categorized participants as boys and girls.^18^ The following categories measured self-reported participant race: White alone, Black alone, Asian alone, other (including multiracial). Participants’ ethnicity was categorized as either Hispanic or non-Hispanic.^18^ In order to be comparable to the Surgeon General’s report^3^ and previous publications,^9,10,19^ investigators combined race and ethnicity to create 4 categories: non-Hispanic White, Hispanic, non-Hispanic Black, and non-Hispanic other (including multiracial participants).

#### Age of Initiation

The PATH study provided a derived variable for participant age in years at each wave, because participant date of birth is not included in the restricted-use data.^18^ The PATH study provided another derived variable to represent the number of weeks between waves that youth participated in.^18^ Participant age was converted from years to weeks and added to the number of weeks between survey dates to give us a more precise estimate of participant age. Lower and upper age bounds were created to identify the age at the last wave when the participant reported nonsusceptibility to cigarette use and the age at the first wave when they reported susceptibility to cigarette use. For participants who did not report initiation of susceptibility to cigarette use over the follow-up period (2014-2017), their upper bound was considered censored. For ever use, past 30-day use, and fairly regular use outcomes, the age of initiation was estimated in the same way as susceptibility based on when the outcome was first reported (2014-2017) for those who become users and the last report of nonuse for those who do not.

### Statistical Analysis 

Secondary analyses of the PATH study restricted-use data sets were conducted^18^ from October 7, 2019, to May 1, 2020, using SAS, version 9.4 (SAS Institute).^20^ A type I error level of 0.05 was used, and all *P* values were 2 sided. All data analyses incorporated sampling weights, 100 balance repeated replicate weights, and a Fay correction factor of 0.3 to account for the PATH complex study design.^18^ Weighted summary statistics (means for continuous variables and proportions for categorical variables) are provided. Weighted interval-censoring survival analysis^21,22,23,24,25^ was implemented to estimate the probability of the age of initiation of susceptibility to cigarette use, ever use, past 30-day use, and fairly regular cigarette use. Hazard functions for each cigarette use outcome overall are reported as cumulative percentages, and 95% CIs are reported using the Turnbull method.^21,22,23,24,25^ Differences in the age of cigarette initiation for each outcome by sex and by race/ethnicity were estimated by fitting weighted Cox proportional hazards regression models to interval-censored data with a piecewise constant function as the baseline hazard function.^21,22,23,24,25^ Hazard functions and 95% CIs are only reported by sex and by race/ethnicity for the outcomes that exhibited statistically significant differences. There was very little missingness in PATH, and the number of participants with missing sex or race/ethnicity are reported in Table 1.

**Table 1.** Demographic Characteristics of the PATH Youth Aged 12-17 Years^a^ Who Were Nonsusceptible to Cigarette Use and Never Cigarette Users at Their First Wave of Study Participation, 2013-2016

Characteristic	Nonsusceptible to cigarette use at first wave of participation			Never cigarette users at first wave of participation		
	Sample No. (n = 11 022)	Estimated national population No. (n = 20 687 132)	Weighted % (SE)	Sample No. (n = 15 776)	Estimated national population No. (n = 29 562 216)	Weighted % (SE)
Wave of entry into study						
Wave 1	7879	14 392 886	69.6 (0.31)	11 769	21 448 494	72.5 (0.18)
Wave 2	1595	3 139 371	15.2 (0.24)	2021	3 984 220	13.5 (0.13)
Wave 3	1548	3 154 875	15.3 (0.34)	1986	4 129 501	14.0 (0.21)
Age, weighted mean (SE)	11 022	20 687 132	13.5 (0.01)	15 776	29 562 216	13.7 (0.01)
Sex						
Boy	5655	10 544 567	51.0 (0.32)	8081	15 056 608	51.0 (0.15)
Girl	5356	10 116 398	49.0 (0.33)	7686	14 484 302	49.0 (0.15)
Missing value	11	26 167	NA	9	21 307	NA
Race/ethnicity						
Hispanic	2218	3 096 942	15.2 (0.20)	4016	5 789 647	19.8 (0.13)
Non-Hispanic Black	1565	2 954 460	14.5 (0.26)	2223	4 180 658	14.3 (0.14)
Non-Hispanic other^b^	1162	2 372 797	11.7 (0.27)	1595	3 221 903	11.0 (0.24)
Non-Hispanic White	5873	11 908 783	58.6 (0.46)	7798	16 104 763	55.0 (0.29)
Missing values	204	354 150	NA	144	265 245	NA

Abbreviation: NA, not available.

^a^ Population Assessment of Tobacco and Health (PATH) Study data reprinted with permission from the United States Department of Health and Human Services.^18^ Restricted file received disclosure to publish: February 10, 2020; February 17, 2020; February 28, 2020; March 06, 2020; May 01, 2020; December 16, 2020.

^b^ Non-Hispanic other indicates non-Hispanic Asian participants, multirace/ethnicity, etc.

## Results

A total of 15 776 youth who were never cigarette users and, among them, 11 022 youth who were nonsusceptible to cigarette use, were included in the study. Among the 11 022 youth who were nonsusceptible to cigarette use, the weighted mean (SE) age was 13.5 (0.01) years, 69.6% (SE, 0.31%) entered the study at wave 1, 58.6% (SE, 0.46%) identified as non-Hispanic White, and 51.0% (SE, 0.32%) were boys (Table 1). Among the 15 776 who were never users of cigarettes at their first wave of PATH participation, the weighted mean (SE) age was 13.7 (0.01) years, 55.0% (SE, 0.29%) were non-Hispanic White, and 51.0% (SE, 0.15%) were boys (Table 1).

Table 2 shows the estimated distribution of the age at initiation of the 4 cigarette use outcomes overall, and eFigure 1 in the Supplement shows these hazard functions. Among youth who were nonsusceptible to cigarette use, by age 13 years, 12.4% were estimated to have become susceptible to cigarette use. By ages 18 and 21 years, these estimates were 46.2% and 61.0%, respectively. Among youth who were never cigarette users, by age 18 years, 24.4%, 16.4%, and 4.3% were estimated to initiate ever, past 30-day, and fairly regular cigarette use, respectively.

**Table 2.** Hazard Functions in Cumulative Percentages of the Age of Initiation of Cigarette Use Outcomes for PATH Youth Aged 12-17^a^

Age, y	Weighted percentage (95% CI) of cigarette use by age^b^			
	Susceptibility to use	Ever use	Past 30-d use	Fairly regular use
12	0	0	0	0
13	12.4 (11.5-13.3)	1.3 (1.1-1.5)	0.5 (0.4-0.6)	0.3 (0.2-0.4)
14	18.6 (17.6-19.7)	2.9 (2.6-3.2)	1.2 (1.0-1.5)	0.6 (0.5-0.8)
15	25.5 (24.2-26.8)	5.2 (4.8-5.7)	2.3 (2.0-2.7)	1.3 (1.1-1.5)
16	31.3 (30.0-32.7)	8.6 (7.9-9.2)	4.3 (3.8-4.8)	2.4 (2.1-2.7)
17	40.1 (38.5-41.8)	15.4 (14.5-16.2)	9.0 (8.2-9.8)	3.3 (2.9-3.8)
18	46.2 (44.3-48.2)	24.4 (22.9-25.9)	16.4 (15.2-17.6)	4.3 (3.9-4.8)
19	50.2 (47.9-52.4)	30.8 (28.8-32.9)	20.7 (18.8-22.6)	5.7 (4.6-6.9)
20	56.2 (49.1-63.3)	33.1 (28.7-37.5)	27.9 (19.6-36.3)	7.0 (5.1-9.0)
21	61.0 (42.9-79.2)	NA	NA	NA

Abbreviation: NA, not available.

^a^ Population Assessment of Tobacco and Health (PATH) Study data reprinted with permission from the United States Department of Health and Human Services.^18^ Restricted file received disclosure to publish: February 10, 2020; February 17, 2020; February 28, 2020; March 06, 2020; May 01, 2020.

^b^ 95% CI indicates Turnbull 95% CI.

Table 3 shows the hazard ratios (HRs) and 95% CIs exploring the differences in the age of initiation of the 4 cigarette use outcomes by sex and by race/ethnicity. We found that boys had a higher risk of initiating ever cigarette use at earlier ages compared with girls (HR, 1.21; 95% CI, 1.08-1.36), as well as past 30-day cigarette use (HR, 1.27; 95% CI, 1.10-1.47). We did not observe statistically significant differences in the age of initiation by sex in susceptibility to cigarette use or fairly regular cigarette use. Non-Hispanic Black youth had 23% less risk (HR, 0.77; 95% CI, 0.68-0.88) to become susceptible to cigarette use at earlier ages compared with non-Hispanic White youth. Non-Hispanic Black (HR, 0.59; 95% CI, 0.49-0.71) and non-Hispanic other (HR, 0.72; 95% CI, 0.59-0.88) youth had a lower risk to initiate ever cigarette use at an earlier age compared with non-Hispanic White youth. Compared with all other racial/ethnic groups, non-Hispanic White youth had a higher risk to initiate past 30-day cigarette use (non-Hispanic Black: HR, 0.64 [95% CI, 0.52-0.77]; non-Hispanic other: HR, 0.77 [95% CI, 0.63-0.95]; and Hispanic: HR, 0.82 [95% CI, 0.69-0.96]) and fairly regular cigarette use (non-Hispanic Black: HR, 0.25 [95% CI, 0.14-0.43]; non-Hispanic other: HR, 0.67 [95% CI, 0.48-0.95]; and Hispanic: HR, 0.64 [95% CI, 0.47-0.87]) at earlier ages than all the other race/ethnicity groups.

**Table 3.** Hazard Ratios (95% CIs) for Different Outcomes of Age of Cigarette Initiation Stratified by Sex and Race/Ethnicity^a^

Characteristic	Susceptibility to use	Ever use	Past 30-d use	Fairly regular use
Sex				
Girls	1 [Reference]	1 [Reference]	1 [Reference]	1 [Reference]
Boys	0.98 (0.91-1.07)	1.21 (1.08-1.36)	1.27 (1.10-1.47)	1.21 (0.97-1.50)
Race/ethnicity				
Non-Hispanic White	1 [Reference]	1 [Reference]	1 [Reference]	1 [Reference]
Non-Hispanic Black	0.77 (0.68-0.88)	0.59 (0.49-0.71)	0.64 (0.52-0.77)	0.25 (0.14-0.43)
Non-Hispanic other^b^	1.08 (0.93-1.25)	0.72 (0.59-0.88)	0.77 (0.63-0.95)	0.67 (0.48-0.95)
Hispanic	0.98 (0.89-1.08)	0.92 (0.80-1.05)	0.82 (0.69-0.96)	0.64 (0.47-0.87)

^a^ Population Assessment of Tobacco and Health (PATH) Study data reprinted with permission from the United States Department of Health and Human Services.^18^ Restricted file received disclosure to publish: February 10, 2020; February 17, 2020; February 28, 2020; March 06, 2020; May 01, 2020.

^b^ Non-Hispanic other indicates non-Hispanic Asian participants, multirace/ethnicity, etc.

Table 4 shows the estimated distribution of the age at initiation for each of the outcomes with statistically significant differences by sex, and eFigure 2 in the Supplement shows the hazard functions. By age 18 years, 27.4% (95% CI, 25.1%-29.6%) of boys and 21.6% (95% CI, 19.8%-23.5%) of girls reported initiation of ever cigarette use. By age 18 years, 19.0% (95% CI, 17.1%-21.0%) of boys and 13.7% (95% CI, 12.2%-15.2%) of girls reported initiation of past 30-day use.

**Table 4.** Hazard Functions in Cumulative Percentages (and 95% CIs) for Age of Cigarette Initiation Outcomes by Sex^a^

Age, y	Initiation of ever cigarette use, % (95% CI)		Initiation of past 30-d cigarette use, % (95% CI)	
	Boys	Girls	Boys	Girls
12	0	0	0	0
13	1.2 (0.8-1.5)	1.5 (1.1-1.8)	0.4 (0.2-0.5)	0.7 (0.5-0.8)
14	2.8 (2.4-3.3)	3.0 (2.6-3.5)	1.1 (0.8-1.4)	1.4 (1.1-1.7)
15	5.0 (4.4-5.6)	5.5 (4.8-6.2)	2.0 (1.5-2.5)	2.7 (2.3-3.2)
16	8.8 (7.9-9.7)	8.4 (7.5-9.3)	4.3 (3.6-5.0)	4.3 (3.7-4.9)
17	16.2 (14.5-17.8)	14.6 (13.3-15.9)	9.4 (8.1-10.8)	8.7 (7.7-9.8)
18	27.4 (25.1-29.6)	21.6 (19.8-23.5)	19.0 (17.1-21.0)	13.7 (12.2-15.2)
19	35.7 (32.3-39.2)	25.9 (23.1-28.7)	24.7 (21.8-27.6)	16.7 (14.4-19.1)
20	NA	31.1 (24.7-37.7)	30.0 (21.7-38.4)	17.9 (12.1-23.8)
21	NA	NA	NA	28.4 (14.4-42.4)

Abbreviation: NA, not available.

^a^ Population Assessment of Tobacco and Health (PATH) Study data reprinted with permission from the United States Department of Health and Human Services.^18^ Restricted file received disclosure to publish: February 10, 2020; February 17, 2020; February 28, 2020; March 06, 2020; May 01, 2020.

Table 5 shows the estimated hazard function of the age at initiation for each of the 4 outcomes by race/ethnicity, and eFigure 3 in the Supplement shows the hazard functions graphically. By age 13 years, 12.7% (95% CI, 11.6%-13.8%) of non-Hispanic White youth, 10.1% (95% CI, 8.6%-11.5%) of Hispanic youth, 10.6% (95% CI, 8.3%-12.9%) of non-Hispanic Black youth, and 15.0% (95% CI, 12.4%-17.5%) of non-Hispanic other youth are estimated to become susceptible to cigarette use.

**Table 5.** Hazard Functions in Cumulative Percentages (and 95% CIs) for Age of Cigarette Initiation Outcomes by Race/Ethnicity^a^

Age, y	% (95% CI)			
	Non-Hispanic White	Hispanic	Non-Hispanic Black	Non-Hispanic other^b^
**Onset of susceptibility cigarette use**				
12	0	0	0	0
13	12.7 (11.6-13.8)	10.1 (8.6-11.5)	10.6 (8.3-12.9)	15.0 (12.4-17.5)
14	19.1 (17.7-20.5)	17.6 (15.7-19.5)	15.9 (13.5-18.2)	19.3 (16.2-22.4)
15	25.9 (24.2-27.7)	25.7 (23.4-28.1)	20.6 (17.9-23.2)	27.1 (23.1-31.0)
16	32.1 (30.2-33.9)	30.8 (28.6-33.0)	26.5 (23.3-29.6)	33.5 (29.2-37.8)
17	41.3 (38.8-43.8)	41.4 (38.8-44.0)	32.5 (28.8-36.2)	41.4 (35.5-47.4)
18	46.0 (43.2-48.8)	49.3 (46.2-52.5)	39.2 (34.7-43.8)	49.9 (44.0-55.8)
19	49.1 (46.2-52.1)	53.8 (50.1-57.6)	44.4 (38.0-50.8)	55.9 (46.9-64.9)
20	57.1 (46.6-67.6)	61.1 (50.2-71.9)	NA	56.6 (41.6-71.6)
21	NA	63.8 (46.8-80.9)	NA	NA
**Initiation of ever cigarette use**				
12	0	0	0	0
13	1.4 (1.2-1.8)	0.9 (0.6-1.3)	1.4 (0.6-1.9)	1.1 (0.5-1.8)
14	3.3 (2.8-3.8)	2.5 (1.8-3.2)	2.3 (1.4-3.2)	2.4 (1.6-3.3)
15	6.0 (5.3-6.6)	5.0 (4.1-5.8)	3.5 (2.4-4.6)	4.3 (2.9-5.7)
16	10.1 (9.1-11.1)	8.1 (6.7-9.5)	5.0 (3.8-6.3)	6.8 (5.1-8.4)
17	17.1 (15.7-18.6)	16.9 (15.0-18.9)	9.5 (7.4-11.6)	11.7 (9.1-14.3)
18	26.2 (24.1-28.3)	25.4 (22.4-28.4)	19.2 (16.2-22.2)	20.8 (15.8-25.7)
19	33.4 (30.0-36.8)	33.0 (29.1-36.9)	21.3 (17.9-24.8)	28.9 (20.8-37.0)
20	33.4 (28.1-38.7)	NA	26.1 (13.2-39.1)	36.5 (14.9-58.1)
**Initiation of past 30-d cigarette use**				
12	0	0	0	0
13	0.6 (0.4-0.7)	0.3 (0.1-0.5)	0.3 (0.1-0.7)	0.7 (0.3-1.1)
14	1.4 (1.0-1.8)	0.9 (0.5-1.2)	0.7 (0.2-1.2)	1.4 (0.7-2.1)
15	2.8 (2.3-3.2)	1.9 (1.3-2.5)	1.2 (0.6-1.8)	2.4 (1.4-3.5)
16	5.1 (4.4-5.8)	3.9 (3.0-4.8)	2.5 (1.5-3.5)	3.6 (2.5-4.7)
17	10.5 (9.3-11.7)	9.4 (7.8-10.9)	6.1 (4.3-7.9)	6.7 (4.5-8.9)
18	17.8 (16.2-19.4)	16.1 (13.5-18.7)	14.3 (11.7-17.0)	14.4 (9.8-17.1)
19	21.8 (19.5-24.0)	19.5 (16.2-22.8)	18.7 (13.0-24.3)	21.4 (15.1-27.7)
20	30.0 (22.0-39.8)	23.0 (12.4-33.6)	18.7 (13.4-24.0)	24.9 (7.5-42.4)
21	30.0 (20.5-39.6)	NA	NA	NA
**Initiation of fairly regular cigarette use**				
12	0	0	0	0
13	0.3 (0.1-0.5)	0.2 (0.1-0.4)	0.2 (0.0-0.3)	0.3 (0.1-0.6)
14	0.8 (0.6-1.0)	0.5 (0.2-0.7)	0.2 (0.0-0.4)	0.7 (0.1-1.3)
15	1.8 (1-4-2.1)	0.9 (0.5-1.3)	0.2 (0.0-0.5)	1.1 (0.3-1.9)
16	3.1 (2.6-3.6)	1.9 (1.3-2.5)	1.0 (0.4-1.6)	1.8 (1.2-2.6)
17	4.5 (3.7-5.2)	2.7 (1.7-3.6)	1.1 (0.5-1.7)	2.5 (1.5-3.4)
18	5.4 (4.8-6.2)	3.9 (2.6-5.3)	1.8 (0.8-2.7)	3.3 (2.1-4.6)
19	6.9 (5.3-8.6)	6.4 (3.4-9.4)	NA	4.4 (2.2-6.6)
20	8.1 (5.6-10.6)	9.9 (1.4-18.5)	NA	10.7 (0-25.2)

Abbreviation: NA, not available.

^a^ Population Assessment of Tobacco and Health (PATH) Study data reprinted with permission from the United States Department of Health and Human Services.^18^ Restricted file received disclosure to publish: February 10, 2020; February 17, 2020; February 28, 2020; March 06, 2020; May 01, 2020.

^b^ Non-Hispanic other indicates non-Hispanic Asian participants, multirace/ethnicity, etc.

By age 18 years, 17.8% (95% CI, 16.2%-19.4%) of non-Hispanic White youth, 16.1% (95% CI, 13.5%-18.7%) of Hispanic youth, 14.3% (95% CI, 11.7%-17.0%) of non-Hispanic Black youth, and 14.4% (95% CI, 9.8%-17.1%) of non-Hispanic other youth reported initiation of past 30-day cigarette use. The largest increase in initiation of past 30-day cigarette use occurred between ages 19 and 20 years among non-Hispanic White youth (8.2%), whereas Hispanic youth (6.7%), non-Hispanic Black youth (8.2%), and non-Hispanic other youth (7.7%) exhibited the largest increase in initiation of past 30-day cigarette use between 17 and 18 years old. By age 20, 8.1% (95% CI, 5.6%-10.6%) of non-Hispanic White youth, 9.9% (95% CI, 1.4%-18.5%) of Hispanic youth, and 10.7% (95% CI, 0%-25.2%) of non-Hispanic other youth reported initiation of fairly regular cigarette use.

## Discussion

To our knowledge, this study is the first to provide prospective estimates for the distribution of the ages of initiation of 4 cigarette outcomes among youth in the US from 2013 to 2017. Age of initiation of the different cigarette use behaviors is an important factor to explore, and we have identified the ages at which youth are most vulnerable to initiate each cigarette use behavior. Because we prospectively estimated the age of initiation of fairly regular cigarette use among youth, we could not find other studies with which to compare our results, because, to our knowledge, no other studies have been conducted to prospectively estimate the age of fairly regular cigarette initiation.

Between 2013 and 2017, 46.2% of youth who were not susceptible to cigarette use became susceptible by the age of 18 in our study. The result was similar to what was found in the results from the 2019 NYTS, which indicated that 45.9% of middle and high school students were susceptible to cigarette use.^7^ In our study, we did not observe any significant difference in susceptibility to cigarette use between girls and boys, which was consistent with the result of the 2019 NYTS survey.^7^ From 2013 to 2017, we observed that by age 18 years, non-Hispanic other youth were most susceptible to cigarette use, followed by Hispanic youth, non-Hispanic White youth, and non-Hispanic Black youth. The 2019 NYTS found similar trends, reporting that 49.l% of Hispanic, 46.2% of non-Hispanic other, 45.2% of non-Hispanic White, and 38.3% of non-Hispanic Black middle and high school students were susceptible to cigarette use.^7^

By age 18, 24.4% of youth initiated ever cigarette use in our study. This percentage was slightly higher than in the NYTS surveys, which indicated that among middle and high school students, 21% in the 2014 to 2016 period had ever used cigarettes.^10^ These differences could be due to the longitudinal nature of our study, whereas the NYTS was cross-sectional across multiple years, or due to reporting incidence in our study vs prevalence in NYTS. The 2019 NYTS survey also reported finding significant differences between the prevalence of ever using cigarettes by sex, with 18.3% of boys reporting ever using cigarette compared with 14.2% of girls.^7^ Our study goes beyond the NYTS results by finding that boys initiate ever cigarette use at earlier ages compared with girls and provides incidence across ages.

We found that non-Hispanic Black and non-Hispanic other youth had later ages of initiation of ever cigarette use compared with non-Hispanic White youth, whereas there were no significant differences observed in the age at initiation of ever cigarette use between non-Hispanic White youth and Hispanic youth. This is different from the NYTS surveys from 2014 to 2017, which found that American Indian and Alaska native youth had the highest prevalence of ever cigarette use (31.4%), followed by native Hawaiian and Pacific Islander youth (29.4%), multiracial youth (24.9%), Hispanic youth (22.2%), non-Hispanic White youth (19.9%), non-Hispanic Black youth (18.0%), and Asian youth (10.3%).^26^ However, PATH did not differentiate between these race/ethnicity categories.

We found that by age 18 years, 16.4% of youth had reported initiation of cigarette use in the past 30 days. In contrast, the results from NYTS surveys reported prevalence of past 30-day cigarette use as (1) 6% in 2014-2016, (2) 5.8% in 2014-2017, and (3) 4.3% in 2019.^7,10,26^ We observed significant differences in the age of initiation of past 30-day cigarette use between boys and girls, suggesting that intervention strategies should start at earlier ages in boys than in girls. The results from the 2019 NYTS survey indicated significant differences by sex in past 30-day cigarette use, with 5.1% among boys and 3.4% among girls.^7^ Although a PATH study of waves 1 to 3 (2013-2016) found that non-Hispanic Black and Hispanic youth had lower odds of initiating past 30-day cigarette use than non-Hispanic White youth,^19^ our study goes beyond by reporting the hazard function of past 30-day cigarette use for each racial/ethnic group.

A similar study^27^ examining the prospective age of e-cigarette initiation among PATH youth never e-cigarette users found that by age 18 years, 50.2% of youth reported susceptibility to e-cigarettes, 41.7% initiated ever use, 23.5% initiated past 30-day use, and 10.3% initiated fairly regular e-cigarette use. These findings suggest that the age of initiation of each behavior was lower for e-cigarettes than for cigarettes. Tests of significance were not performed, but these findings add to the body of literature on e-cigarettes as the most popular tobacco product among youth. Another study regarding the age of cigar product initiation among youth found that by age 18 years, 21.1% initiated ever use of any cigars and 11.3% initiated past 30-day use of any cigars,^28^ which is lower than the estimates provided for cigarette initiation. A recent national study found that past 30-day cigar use surpasses past 30-day cigarette use^7^; more research is needed to determine the reasons behind these findings.

A recent repeated cross-sectional study examined whether the age of cigarette initiation has changed from adolescence (<18 years) to early adulthood (18-23 years) between 2002 and 2018.^29^ That study found that the proportion of ever cigarette initiation occurring in early adulthood more than doubled from 20.6% in 2002 to 42.6% in 2018.^29^ Additionally, a recent study^30^ of PATH youth aged 26 to 34 years (who would have been aged 12 to 17 years in the early 2000s) that examined the recalled age of cigarette initiation found that the cumulative proportion of cigarette initiation was 85.8% by age 18 years. This finding is subject to recall bias; however, our results for youth aged 12 to 17 years between 2013 and 2017 showed that only 24.4% of cigarette initiation occurred by age 18 years, which could be explained by the shifting of cigarette initiation to later ages. Although the age of cigarette initiation may be shifting toward early adulthood rather than adolescence, suggesting that cigarette interventions should be implemented among young adults, our study suggests that a high amount of cigarette initiation still occurs among youth. The age of initiation shifting toward later ages could be due to the introduction of e-cigarettes,^7^ successful interventions targeted toward youth,^31,32,33^ or other possible explanations. However, a recent article^34^ shows that the introduction of e-cigarettes was followed by a slowing decline in past 30-day cigarette use and acceleration in the ever cigarette use trend between 2004 and 2018. In addition, another article^35^ indicates that there was actually no decline in cigarette use because of the introduction of e-cigarettes. More research is needed to determine which of these explanations holds.

There is considerable evidence that earlier ages of cigarette initiation are associated with greater nicotine dependence and greater exposure to nicotine,^36^ which can result in adverse health outcomes, including altering brain development among youth^37^ and respiratory illnesses.^38^ Previous research has established that nicotine-dependent youth are less likely to want to quit using cigarettes, and youth are uniquely vulnerable to developing nicotine addiction, even at low levels of nicotine use.^39^ Interventionists and the public can use our results to identify the particular ages at which the campaigns may be most effective to implement to prevent youth from becoming susceptible to cigarette use and engaging in harmful cigarette use behaviors, such as past 30-day and fairly regular cigarette use.

### Strengths and Limitations

Strengths of this study include using a nationally representative data set and the longitudinal prospective analysis of cigarette initiation outcomes across PATH waves 1 to 4. One of the limitations of our study is that we depended on self-reported data for the cigarette use outcomes to estimate the age of initiation because asking participants the exact date they initiated cigarettes is unrealistic.

## Conclusions

To our knowledge, this cohort study is the first to provide prospective estimation of the age of initiation of susceptibility, ever, past 30-day, and fairly regular cigarette use among youth in the US between 2013 and 2017. This study provides windows of opportunity by identifying the ages of 4 cigarette use outcomes in which never users are most likely to initiate cigarette use, including by sex and by race/ethnicity. The results from our study suggest that among those who initiated cigarette use, the onset of the 4 cigarette outcomes occurred for the majority of youth before they were aged 18 years (between 16 and 18 years). Because the age of initiation of cigarette use was different by sex and by race/ethnicity, it is important to tailor preventive interventions considering those differences. Boys and non-Hispanic White youth had an increased risk of initiating cigarette use at earlier ages, suggesting that tobacco control interventions should focus on these vulnerable subgroups at earlier ages. In December 2019, the government changed the legal age to purchase tobacco products from 18 to 21 years.^40^ Though the law did not affect our results, this study provides additional evidence to support the new regulation and can serve as a reference point for age of initiation of cigarette use in future studies after implementation of the Tobacco 21 campaign.^40^

## Notes

Supplement. **eFigure 1.** Estimated Age of Initiation of Cigarette Use Outcomes **eFigure 2.** Estimated Age of Initiation of Cigarette Use Outcomes by Sex **eFigure 3.** Estimated Age of Initiation of Cigarette Use Outcomes by Race/Ethnicity
