# Transcription Errors in Numbers Reported

**DOI:** 10.1001/jamanetworkopen.2021.10182

**Published:** 2021-04-09

In the Original Investigation, “Age at Initiation of Cigarette Use in a Nationally Representative Sample of US Youth, 2013-2017,”^1^ published February 26, 2021, in *JAMA Network Open*, incorrect values were reported because of transcription errors before the manuscript was submitted. The errors affect numbers reported in Tables 2, 4, and 5, and the Results section, 2 sentences in the Discussion, 2 sentences in the Abstract, and eFigure 3 in the Supplement. The conclusions based on the results were not affected. The article has been corrected.^1^
