# Supplementary material for: Age at Initiation of Cigarette Use in a Nationally Representative Sample of US Youth, 2013-2017
Source: JAMA Netw Open. 2021 Feb 26;4(2):e210218. doi: 10.1001/jamanetworkopen.2021.0218 (PMC7910812; doi:10.1001/jamanetworkopen.2021.0218)
Supplement: Supplement. — eFigure 1. Estimated Age of Initiation of Cigarette Use Outcomes eFigure 2. Estimated Age of Initiation of Cigarette Use Outcomes by Sex eFigure 3. Estimated Age of Initiation of Cigarette Use Outcomes by Race/Ethnicity [file jamanetwopen-e210218-s001.pdf]

Supplementary Online Content

Pérez A, N'hpang RS, Callahan E, et al. Age at initiation of cigarette use in a nationally representative sample of US youth, 2013-2017. *JAMA Netw Open*. 2021;4(2):e210218. doi:10.1001/jamanetworkopen.2021.0218

eFigure 1. Estimated Age of Initiation of Cigarette Use Outcomes

eFigure 2. Estimated Age of Initiation of Cigarette Use Outcomes by Sex

eFigure 3. Estimated Age of Initiation of Cigarette Use Outcomes by Race/Ethnicity

This supplementary material has been provided by the authors to give readers additional information about their work.

© 2021 Pérez A et al. *JAMA Network Open.*

eFigure1. Estimated Age of Initiation of Cigarette Use Outcomes

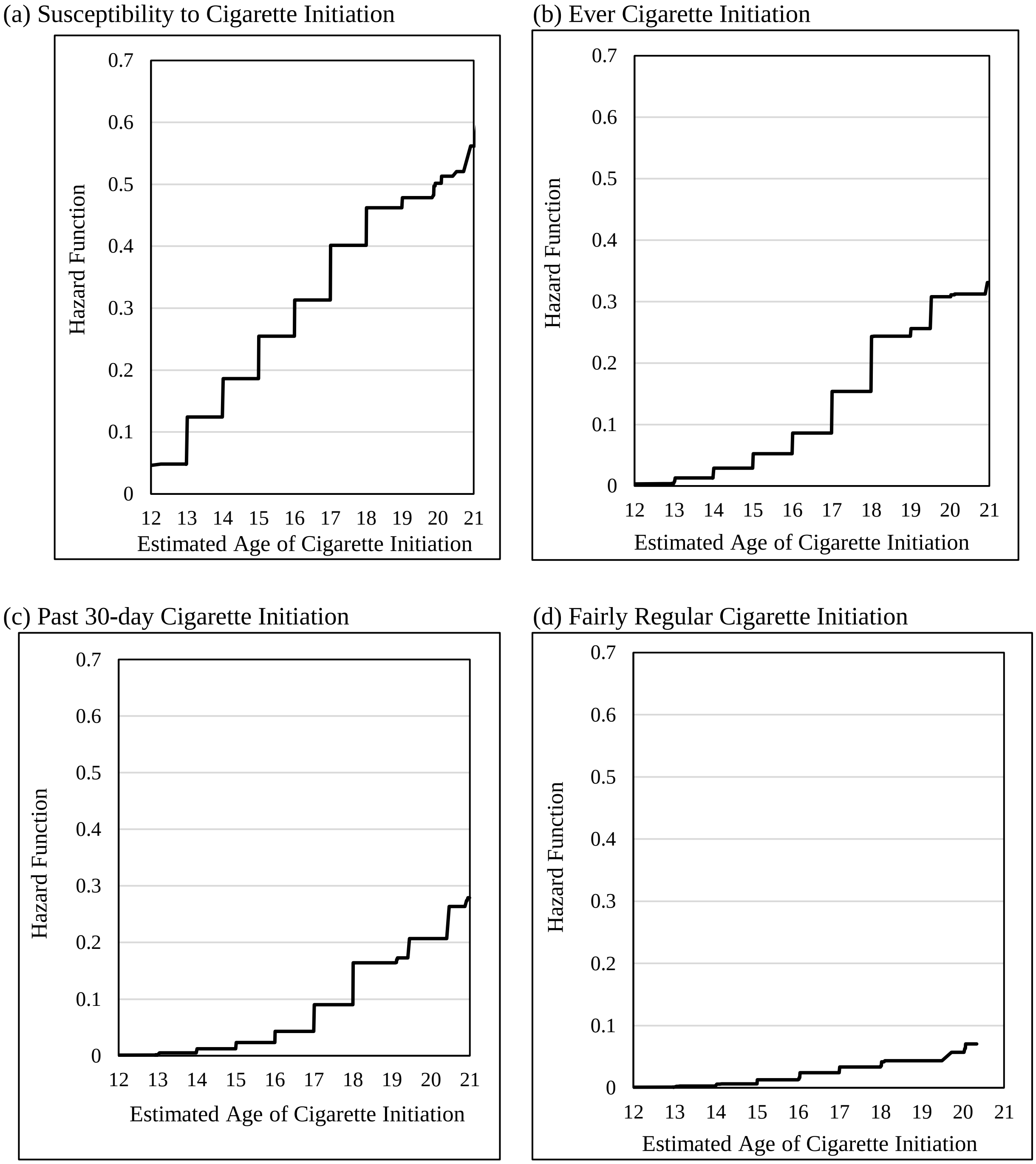

© 2021 Pérez A et al. *JAMA Network Open.*

eFigure2. Age of Initiation of Cigarette Use Outcomes by Sex

(a) Ever Cigarette Initiation

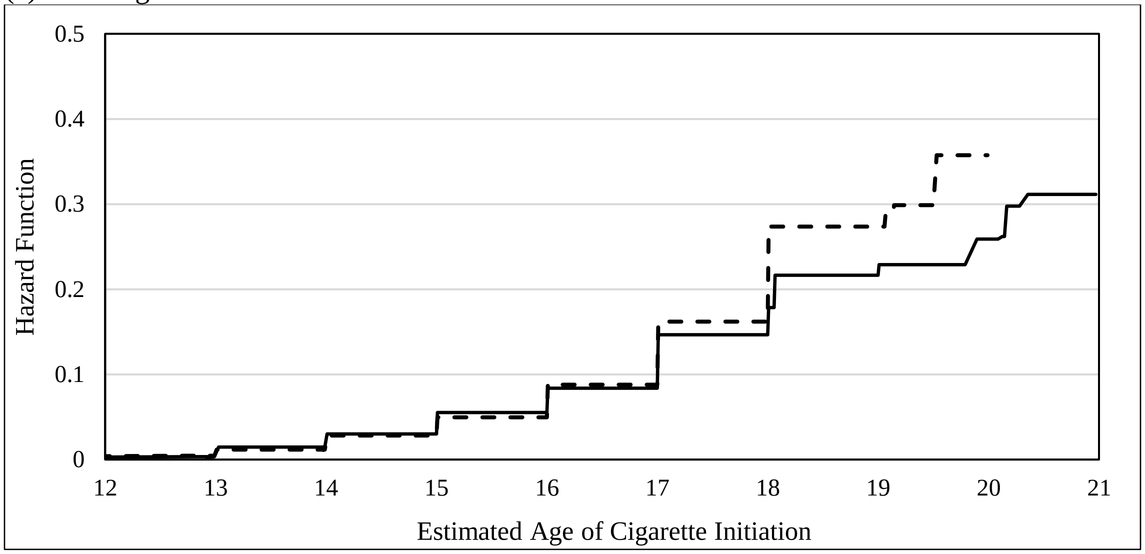

(b) Past 30-Day Cigarette Initiation

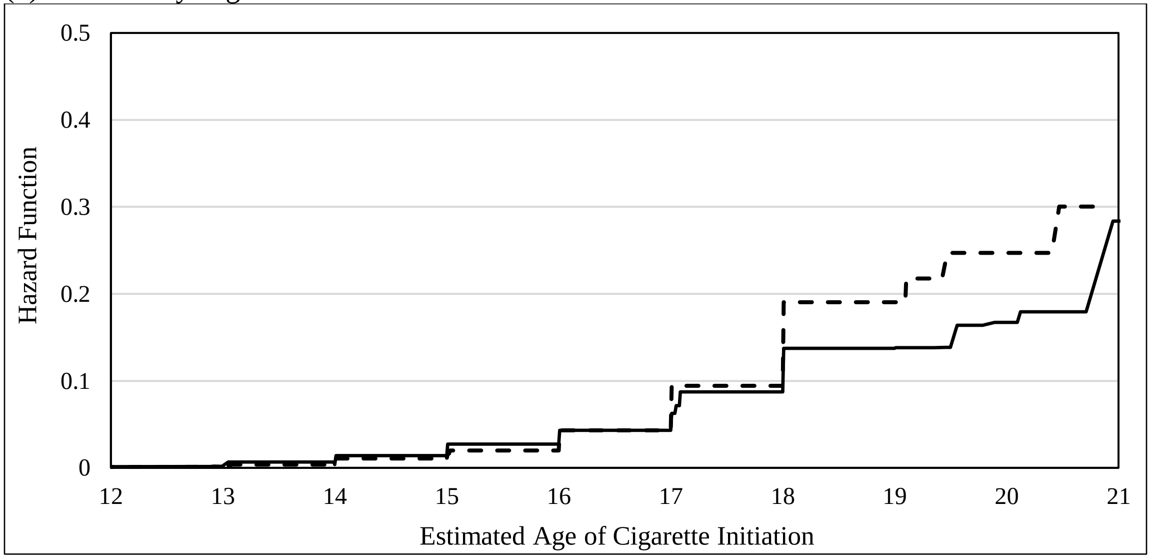

- - Male —— Female

© 2021 Pérez A et al. *JAMA Network Open.*

eFigure3. Age of Initiation of Cigarette Use Outcomes by Race/Ethnicity.

(a) Susceptibility to cigarette initiation

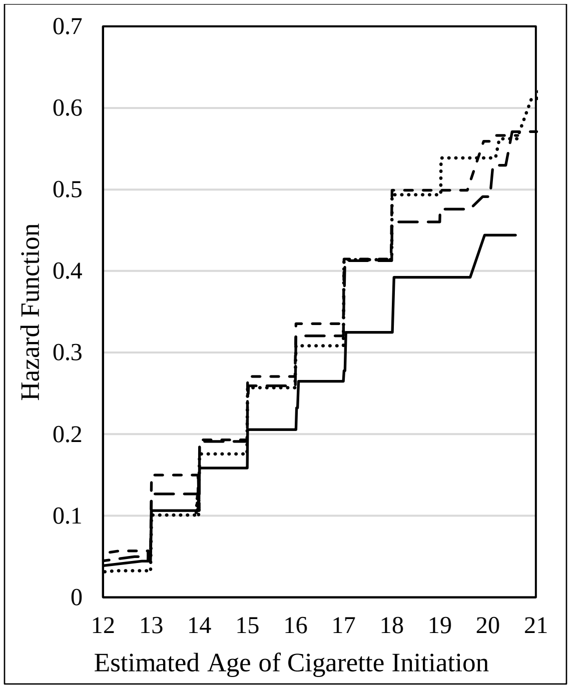

(b) Ever Cigarette Initiation

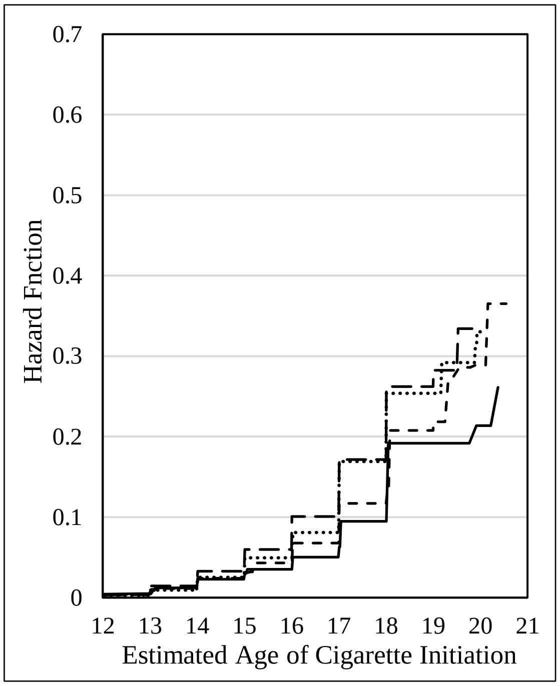

(c) Past 30-day Cigarette Initiation

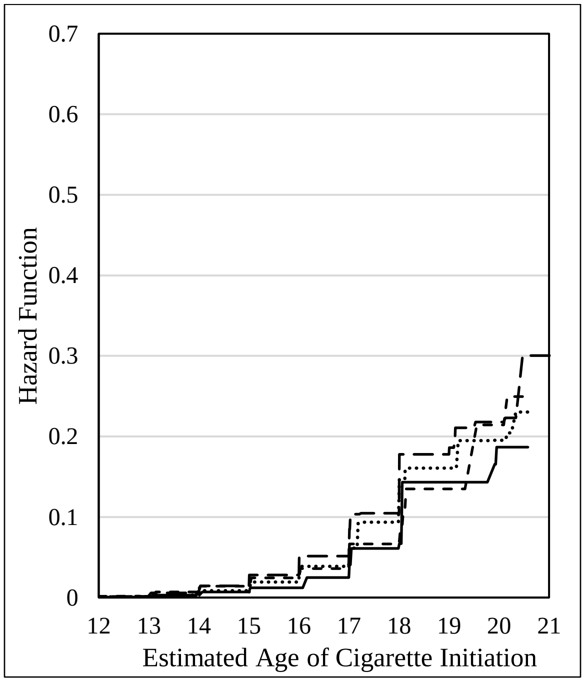

(d) Fairly Regular Cigarette Initiation

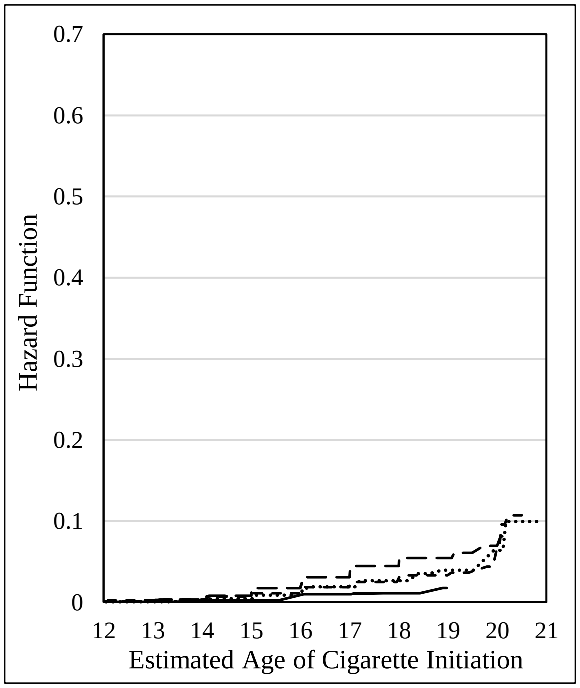

Hispanics — Non-Hispanic Blacks — Non-Hispanic Asians/Others — Non-Hispanic Whites

© 2021 Pérez A et al. *JAMA Network Open.*
